# A qualitative study on barriers and facilitators of COVID-19 vaccination during pregnancy among pregnant women in Malaysia

**DOI:** 10.1371/journal.pone.0329895

**Published:** 2025-08-08

**Authors:** Muhammad Hafiz Mohamed Pauzi, Azidah Abdul Kadir, Rosediani Muhamad, Zainab Mat Yudin

**Affiliations:** 1 Department of Family Medicine, School of Medical Sciences, Universiti Sains Malaysia, Kota Bharu, Kelantan, Malaysia; 2 Outpatient Family Health Clinic, Universiti Sains Malaysia Specialist Hospital, Kubang Kerian, Kelantan, Malaysia; 3 Medical and Basic Dental Sciences Unit, School of Dental Sciences, Universiti Sains Malaysia, Kubang Kerian, Kelantan, Malaysia; University of Ferrara, ITALY

## Abstract

**Background:**

COVID-19 vaccination is crucial for pregnant women to protect against severe complications during pregnancy. However, vaccine hesitancy and refusal can affect acceptance rates among this vulnerable group, posing risks to both maternal and fetal health. This research was conducted to explore the barriers and facilitators influencing COVID-19 vaccination among Malaysian pregnant women.

**Methods:**

This qualitative study adopted a hermeneutic phenomenological design. Nineteen pregnant women receiving antenatal care at three clinics in Kelantan between February 2024 and June 2024 were purposively selected to participate in in-depth interviews. The interviews were audio-recorded, transcribed verbatim, and analyzed using NVivo software alongside manual coding. Thematic analysis, incorporating both inductive and deductive approaches, was conducted to identify key themes.

**Results:**

Most pregnant women expressed hesitancy toward COVID-19 vaccination during pregnancy due to various concerns. Our findings identified four key themes as barriers to vaccination: (i) Fear of side effects, (ii) Negative social influences, (iii) Perception of returning to normalcy, and (iv) Vaccine scepticism and prejudice. Conversely, three themes emerged as facilitators of vaccine acceptance: (i) Concerns about the effects of COVID-19 infection, (ii) Trust in the vaccine, and (iii) Personal and shared vaccination experiences.

**Conclusion:**

This study identified key factors influencing COVID-19 vaccine hesitancy. These findings highlight the importance of addressing both personal and social dimensions of vaccine hesitancy. Public health interventions should focus on building trust, countering misinformation, and leveraging positive community experiences to encourage vaccine uptake, particularly in populations where hesitancy remains a concern.

## Introduction

COVID-19 vaccination is crucial for pregnant women to prevent severe complications associated with COVID-19 infection. Pregnant women are at higher risk of adverse outcomes, including preeclampsia, heart failure, preterm birth, fetal distress, and premature rupture of the membrane [1,2], and highly recommended for COVID-19 vaccination by the World Health Organization (WHO) [3]. Nevertheless, despite these recommendations, vaccine hesitancy remains a significant challenge, affecting vaccine acceptance rates and potentially jeopardizing both maternal and fetal well-being. Vaccine hesitancy is defined as a delay in accepting or outright refusal of vaccines despite their availability [4]. The WHO has identified vaccine hesitancy as a major public health threat that requires strategic intervention to prevent outbreaks of vaccine-preventable diseases (VPD) [4]. WHO has announced vaccine hesitancy as one of the major public health threats, and this issue should be strategically tackled to prevent the rise of VPD [5].

Globally, according to a cross-sectional survey in 16 countries, the COVID-19 vaccine acceptance rate among pregnant women ranges between 28.8% and 84.4% [6]. Skjefte et al. found that India, the Philippines, and all Latin American countries had higher COVID-19 vaccine acceptance rates; conversely, Russia, the United States, and Australia had lower acceptance rates [6]. Multiple reasons for vaccine hesitancy were identified in pregnant women’s perspectives, and this vaccine hesitancy is dynamic. Reasons identified in previous studies including safety concerns, lack of confidence, misinformation/lack of knowledge, trust issues with healthcare providers, cultural/social norms [7–9].

A multi-center study showed a high maternal COVID-19 vaccine acceptance rate of 77.1% among urban pregnant women in Malaysia [10]. Most existing studies on COVID-19 vaccination among pregnant women in Malaysia were conducted during the pandemic or the initial vaccine rollout, with a predominant focus on quantitative data. However, as COVID-19 transitions from a pandemic to an endemic phase, vaccine perceptions may evolve due to changing circumstances. The Health Belief Model (HBM) and Theory of Planned Behaviour (TPB) were two common frameworks used in examining the vaccine uptake and hesitancy either for quantitative or qualitative studies [11–16]. Qualitative studies can provide in-depth insights into the personal experiences, beliefs, and concerns of pregnant women regarding COVID-19 vaccination. This helps identify specific barriers to vaccine uptake that may not be captured by quantitative surveys.

This study aims to explore the barriers and facilitators to COVID-19 vaccination among Malaysian pregnant women in a suburban area (Kelantan state). The findings from this study will help to give more insight into the acceptance of vaccines among pregnant women, who are a vulnerable group to getting infections during disease outbreaks with either different COVID-19 variants or new emerging diseases. Furthermore, it will assist healthcare providers (HCPs) in tackling the issues of vaccine hesitancy among pregnant women in this region.

## Materials and methods

### Study design

A phenomenological research design was applied to explore Malaysian pregnant women’s barriers and facilitators to COVID-19 vaccination during pregnancy. In-depth semi-structured interviews were conducted in Malay language, face-to-face on the field, with open-ended and exploratory questions to gather non-numerical data. The researcher’s team consists of three family medicine specialists (FMS) and one FMS in training. Two of the FMS are known experts in conducting qualitative research for more than 10 years. The interviews were conducted by single interviewer (principal researcher) which was the FMS in training. The FMS in training had undergone training in qualitative research and the initial two interviews were conducted under supervision of the FMS to ensure good and structured interviews. The principal researcher maintained self-reflexivity throughout the research process to avoid personal assumptions about participants’ descriptions. A topic guide was created based on the theoretical frameworks of HBM and the TPB. HBM suggests that individuals’ decisions to engage in health-related behaviors (getting vaccinated) and TPB posits that an individual’s intention to be vaccinated [17–19]. Using both HBM and TPB provides a comprehensive framework for exploring complex reasons behind vaccine hesitation among pregnant women regarding COVID-19 vaccines in Malaysia. This approach helps identify specific psychological and social factors driving vaccine hesitancy among this demographic group. The interviews were also conducted in comfy meeting rooms at local health clinics, antenatal clinics, and wards to ensure privacy. Each participant received RM50 (approximately USD12) as a token of appreciation for their voluntary enrolment. The honorarium may appear modest in some contexts; however, it is considered appropriate and adequate within the Malaysian setting. The compensation was determined in line with local ethical guidelines and norms to appreciate participants’ time without exerting undue influence on their decision to participate.

### Participants and setting

Participants were pregnant women attending three antenatal clinics in Kelantan, including Universiti Sains Malaysia Specialist Hospital, Bachok Health Clinic, and Rantau Panjang Health Clinic. To focus on those who experience COVID-19 vaccination activity either receiving, refusing or hesitating, a purposeful sample was selected from clinics. Key informants (chief staff nurses) were used to identify suitable participants. The inclusion criteria included Malaysian citizens and pregnant women aged 18 and above who could comprehend and read in Malay language. The exclusion criteria were pregnant women who had acute symptoms of labor or any unstable conditions (uncontrolled hypertension, preeclampsia, etc.), pregnancy with intrauterine death, or any psychiatric conditions. We provided research information together with written consent for those agreeable in participating in the study.

Interviews were conducted over a 16-week from February 29th to June 29th, 2024, and each interview lasted between 30 and 60 minutes. Data collection was discontinued when data saturation was reached.

### Analysis

All interviews were audio-recorded and transcribed verbatim. The data collections were initially managed using the computer software NVivo; subsequently, by-hand table work was employed using Microsoft Word software. The transcriptions were checked by participants and read thoroughly and repeatedly by all researchers to ensure rigor and trustworthiness. The interviews were conducted until the data was saturated, which is described as no more new information, themes or any insights relevant to the research questions were obtained. The principal researcher conducted the first five interviews, which were later transcribed verbatim. Then, a meeting was arranged with other researchers to identify and organize the themes and subthemes. Subsequent interviews were carried out until no new insights were gathered by the 16th interview. However, three more interviews were conducted to ensure that data saturation had been reached, after which data collection was discontinued. Discussions were held frequently to refine and triangulate the findings. The researchers also look into the previous findings in other studies to ensure no new study insights need to be explored. Thematic analysis with inductive and deductive approaches was employed to ensure thoroughness and minimize bias.

### Rigor and trustworthiness

Prolonged engagement was applied during the in-depth interviews by the main researcher to build rapport with the participants to obtain authentic results. The research process, which included the data collection and analysis details, was fully described (audit trail) and evaluated by all researchers (peer review), including a researcher whose expertise in qualitative research. The transcriptions and interpretations were checked by the participants (member checking) to ensure rigor and trustworthiness.

### Ethical approvals

This project was approved by the Human Research Ethics Committee (JEPEM), Universiti Sains Malaysia (protocol number: USM/JEPeM/22050297) and Medical Research & Ethic Committee (MREC), Ministry of Health Malaysia (protocol number: 22–01456-AQA).

## Results

Nineteen Malay pregnant women, aged between 18 and 44 years participated in this study (Table 1). Most participants had received secondary or tertiary education, and most were housewives in the third trimester of pregnancy. Eight participants were primigravida and had received COVID-19 vaccination before pregnancy. The majority were hesitant about COVID-19 vaccination during pregnancy and only three participants agreed to receive booster vaccination during the current pregnancy. Hesitant means they might consider taking the vaccination later under certain circumstances but would need to reconsider it in the future. Thematic findings were presented in two main themes (Table 2), the barriers to COVID-19 vaccination during pregnancy and the facilitators of COVID-19 vaccination during pregnancy. Fear of the side effects, negative social influences, perception returning to normalcy, and vaccine skepticism and prejudice were the emerging themes for barriers to COVID-19 vaccination during pregnancy. The themes that emerged from facilitators of COVID-19 vaccination during pregnancy were concerns about the effects of COVID-19 infection, trust in the vaccine, and personal and shared vaccination experiences.

**Table 1:** Participant demographic information.

Pseudonym	Age	Education level	Employment	Gravida	Trimester	COVID-19 Vaccine preference during pregnancy
P1	21	Tertiary	Self-employed	1	Second	Refuse
P2	38	Tertiary	Staff Nurse	1	Second	Refuse
P3	18	Secondary	Housewife	2	Second	Accept
P4	28	Secondary	Housewife	2	Third	Refuse
P5	21	No formal education	Housewife	3	Third	Hesitant
P6	20	Secondary	Housewife	2	Third	Accept
P7	42	Tertiary	Staff Nurse	4	Second	Accept
P8	24	Tertiary	Housewife	1	Third	Hesitant
P9	28	Primary	Housewife	5	Third	Hesitant
P10	33	Tertiary	Optometrist	1	Third	Accept
P11	33	Tertiary	Housewife	2	First	Accept
P12	36	Tertiary	Housewife	1	First	Hesitant
P13	37	Tertiary	Housewife	3	Third	Accept
P14	27	Tertiary	Housewife	1	Third	Refuse
P15	27	Tertiary	Accountant	1	Third	Refuse
P16	44	Secondary	Housewife	2	Second	Refuse
P17	27	Secondary	Housewife	1	Third	Hesitant
P18	31	Secondary	Housewife	4	First	Hesitant
P19	30	Tertiary	Housewife	3	Third	Accept

**Table 2:** Summary of findings.

Barriers to COVID-19 vaccination during pregnancy
1. *Fear of the side effects*
a. Concern about harm to mother and baby b. Previous unpleasant side effects
2. *Negative social influences*
a. Social media’s shaping of perceptions b. Influence from family and peers
3. *Perception returning to normalcy*
4. *Vaccine scepticism and prejudice*
a. Conspiracy theory b. Vaccine inefficacy
**Facilitators of COVID-19 vaccination during pregnancy**
1. *Concerns about the effects of COVID-19 infection*
a. Perceived susceptibility b. Perceived severity
2. *Trust in he vaccine*
a. Protection from COVID-19 infection b. Perceived safety
3. *Personal and shared vaccination experiences*

### Barriers to COVID-19 vaccination during pregnancy

#### 1. Fear of the side effects.

**a) Concern about harm to mother and baby**

The majority of participants voiced their worries about the safety of maternal COVID-19 vaccines to their unborn babies, citing concerns about the possibilities of congenital defects, intrauterine development problems, miscarriage, and premature birth that may occur during perinatal or after-delivery periods. These participants also described their fears of accepting the COVID-19 vaccination during pregnancy due to the lack of trust in the COVID-19 vaccine and its potential adverse effects on themselves in the future. Furthermore, participants also expressed their concerns about maternal COVID-19 vaccine side effects as it is a novel vaccine.

*“To the child, if we take the vaccine... I’m worried it will cause physical defects, growth retardation... any illness after childbirth. I’m worried about my baby’s health.”* (P1, second trimester)

*“I fear there’s a problem with pregnancy. I’m afraid of miscarriage... The effect (of vaccine) on my baby... baby born premature or miscarriage.”* (P4, third trimester)

*“I’m concerned about the side effects especially when I am pregnant... because there was no (COVID-19) vaccine previously, COVID-19 vaccine is a new vaccine.”* (P11, first trimester)

**b) Previous unpleasant side effects**

Several participants described their previous vaccination experiences as unpleasant, making them apprehensive about receiving the vaccine or subsequent booster vaccination. One participant feared receiving the booster vaccine would cause more severe side effects since; after receiving two doses, she felt her immunity had been curtailed.

*“I’ve already had the experience of taking dose 1 and dose 2... my antibodies seem to be weak... I’m afraid to take this booster because I’m afraid it will get worse.”* (P1, second trimester)

Another participant mentioned her booster vaccination experience was similar to her symptoms when she contracted a COVID-19 infection in the past, leading to an unpleasant experience and refusal of another booster vaccine.

*“Because when I received the booster jab, I felt like I had the COVID-19 infection symptoms again. I lost my taste sensation. I just recovered from COVID-19 infection last month, and I started to have a fever, body ache... so I think for the fourth dose I won’t take.”* (P2, second trimester)

#### 2. Negative Social Influences.

**a) Social media’s shaping of perceptions**

Participants had received negative information about vaccine side effects from social media as everyone has been living in a digital era, eliciting fear of safety for their unborn babies.

*“I am afraid the baby has problems, because I get a lot from Facebook, saying the effect of the vaccine on the baby, like this and like that.”* (P4, third trimester)

*“Because I’ve read, when I joined a forum (social media) of pregnant mothers, someone told in the forum that she experienced a miscarriage after being vaccinated. Yes, read that in the early pregnancy, the risk is quite high.”* (P11, first trimester)

**b) Influence from the family and peers**

The negative narratives from surrounding people have impacted pregnant women’s decisions on COVID-19 vaccination. Many participants reported their friends and families claimed that the vaccine may worsen people’s general health rather than provide healthier lives.

*“They (other people) told me that if we get the vaccine, there will be many problems, we’ll get a disease, maybe it will get worse... people say it’s not getting better but getting worse... Our health will get worse.”* (P6, third trimester)

*“My husband doesn’t allow (to accept vaccine) if I’m pregnant. One obstacle is the vaccine, then my husband doesn’t allow it. My family doesn’t allow it... That’s it. There is also a friend, who said there is no necessity to take the vaccine, it will cause problems to the baby and myself.”* (P4, third trimester)

#### 3. Perception returning to normalcy.

Almost all participants mentioned the current situation as normal since there was a lack of reported new cases of COVID-19 infections, described as a common flu and used the term “return to normal”.

*“It’s like a normal matter nowadays, because we don’t hear much about people having COVID symptoms. After that, those infected with COVID-19 are asymptomatic. Some people do get COVID but the symptoms are not as bad as we heard before, because many people have been vaccinated.”* (P2, second trimester)

*“COVID-19 infection has become influenza; it is like a common cold.”* (P11, first trimester)

*“The current situation is normal because we’re accustomed to it. There’s nothing to worry about. Return to normal.” (P14, third trimester)*

#### 4. Vaccine skepticism and prejudice.

**a) Vaccine inefficacy**

Participants expressed their lack of confidence in the maternal COVID-19 vaccine due to the perceived lack of vaccine efficacy as the vaccine was new and had not been previously utilized.

*“Before this, the COVID-19 vaccine didn’t exist, like Pfizer, Sinovac, and AstraZeneca have never been used before. I don’t even know if it can reduce (infection) or not.”* (P11, first trimester)

*“Even though we’re vaccinated, I was still infected with COVID-19. So, I feel like... I don’t know what to say... I’m not sure if it will be effective.”* (P15, third trimester)

**b) Conspiracy theory**

Participants reported that the COVID-19 vaccine contained a microchip that would be inserted into the human body for hidden agenda reasons. That belief brought skepticism and prejudice toward the COVID-19 vaccine and hindered its acceptance among people.

*“Because he (my husband) is afraid... he’s not afraid of the medicine, he’s afraid because people say the vaccine has a chip... a chip is inserted into our body.”* (P5, third trimester)

*“Some people mention there is a prohibited item (microchip) in it (COVID-19 vaccine). I don’t know where that vaccine comes from.”* (P4, third trimester)

Furthermore, one participant questioned the emergence of the COVID-19 virus and vaccine and she thought that COVID-19 vaccination was a planned hidden agenda designed to make humans experimental subjects.

*“Why did they make the vaccine? Why did COVID-19 come out? So there must be a possibility that this was all planned. The benefits of the vaccine for pregnant women, personally, there’s no such thing... because maybe the government just wants to give it to pregnant women but doesn’t know about the side effects. Maybe the government wants to give it to make us lab rats.”* (P8, third trimester)

### Facilitators of COVID-19 vaccination during pregnancy

#### 1. Concerns about the effects of COVID-19 infection.

**a) Perceived susceptibility**

The significant theme discerned was participants felt they were susceptible to COVID-19 infection compared to nonpregnant women. Therefore, they needed COVID-19 vaccination to protect themselves as well as their unborn babies.

*“Expectant mothers are not as healthy as nonpregnant women. Their antibodies are weaker. We’re two bodies, we need to think about our baby too.”* (P9, third trimester)

*“To prevent pregnant mothers from COVID-19 infection, maybe even if it can’t prevent it (COVID-19 infection), it can still reduce the risk of getting infected with severe COVID-19 infection. Because pregnant mothers are easily affected by any illnesses.”* (P8, third trimester)

**b) Perceived severity**

Participants expressed that they and their unborn babies were more prone to contract severe COVID-19 complications such as chronic lung illnesses, premature delivery, fetal abnormalities, and death if they were getting infected with COVID-19 infection.

*“Maybe give birth earlier than the expected date (if we get a COVID-19 infection).. if we give birth too early the baby is not mature enough and can’t survive. Then, if we get infected with COVID-19 while pregnant, like what happened to singer Siti Sarah.. she died while pregnant. I’m afraid to be like her. It (COVID-19 infection) will affect the baby too. I’m scared of dying during pregnancy.”* (P5, third trimester)

*“COVID-19 can cause death sometimes... it can cause chronic diseases... So I’m worried if we are pregnant and get infected with COVID-19, it will be more dangerous. COVID-19 can affect my baby... chronic illness or my baby’s lungs become chronic.”* (P1, second trimester)

#### 2. Trust in the vaccine.

**a) Protection from COVID-19 infection**

Participants believed the COVID-19 vaccine during pregnancy would protect themselves and their unborn babies from COVID-19 infection. Additionally, the COVID-19 vaccine received by pregnant women would provide vertical immunity to their unborn babies.

*“To protect myself, and my baby too... It’s important during pregnancy as it prevents us from getting the COVID-19 infection.”* (P5, third trimester)

*“Pregnant women will have stronger antibodies after vaccination. Can prevent illnesses... or COVID-19 infection... the babies will get immunity (protection).”* (P1, second trimester)

**b) Perceived safety**

Participants also felt safe with the COVID-19 vaccine as they believed in the health benefits of the COVID-19 vaccine to themselves and their unborn babies after receiving the vaccination.

*“It was okay... I felt safe taking this vaccine.”* (P6, third trimester)

*“I felt safe because I got vaccinated after delivery and it was uneventful. Perhaps, it’s good for pregnant women.”* (P3, second trimester)

#### 3 Personal and shared vaccination experiences.

Most participants experienced mild side effects after the COVID-19 vaccination during pregnancy, leading them to the acceptance of maternal COVID-19 vaccine. They described the experience as pleasant and uneventful.

*“I got vaccinated during my first pregnancy... It was okay... Personally, this vaccine was incredibly good. It was uneventful. I felt healthier with this vaccine. It’s good based on my vaccination experience.”* (P6, third trimester)

*“So far, Alhamdulillah, there was no problem, because I’ve taken two doses. I mean all is fine. My experiences were fine.”* (P9, third trimester)

The family members’ positive vaccination experiences observed by the participants influenced them to accept the vaccine. One participant observed that the COVID-19 vaccine effectively broke the chains of transmission among her family members.

*“My brother is a soldier; his wife is pregnant. He went to a course and contracted COVID-19. His wife is pregnant with a second child, and she was vaccinated with 2 doses, Alhamdulillah, it doesn’t spread to his wife.”* (P10, third trimester)

## Discussion

Pregnant women expressed that their decision to receive the COVID-19 vaccine was primarily influenced by concerns about their own health and the well-being of their unborn child. Factors such as the perceived benefits of vaccination in protecting against severe illness, reassurance from healthcare providers, and recommendations from health authorities served as key facilitators in their decision-making process. Conversely, fears regarding potential side effects, uncertainty about long-term effects on fetal development, and mixed information from various sources acted as barriers to vaccine acceptance.

Despite these considerations, many pregnant women remained indecisive about vaccination in the post-pandemic period. This hesitation was driven by a reduced perceived threat of COVID-19, skepticism about the continued necessity of vaccination, and lingering doubts fueled by misinformation or conflicting advice. As a result, their decision to accept or refuse the vaccine remained complex and highly individualized.

### Barriers & implications for practice and policy

From our study, we found barriers to COVID-19 vaccination during pregnancy stem from several key concerns including fear of the side effects, negative social influences, perception returning to normalcy, and vaccine skepticism or prejudice. Being afraid to side effects, perception returning to normalcy and vaccine prejudice are aligned with constructs from the HBM, particularly perceived barriers and low perceived susceptibility [11,13–17]. Conversely, having negative social influences was described under the subjective norm in TPB [12–15,18,19]. Policymakers should implement educational initiatives to address misinformation and fear surrounding COVID-19 vaccination during pregnancy. Fear of side effects, including potential harm to both mother and baby, as well as previous unpleasant vaccine experiences, contribute to hesitancy [6,20–26]. Surprisingly, a qualitative study in Bristol, UK, during the first wave of the pandemic found that participants showed a variety of responses on vaccine trials on pregnant women, ranging from feeling it was important but were unwilling to volunteer to mention vaccine trials should not be conducted, and some were uncertain about it, mainly due to fear of the unknown vaccine side effects [27]. Several studies had shown that pregnant women demonstrated similar adverse effects of the COVID-19 vaccine as nonpregnant women [28,29]. Pregnant women expressed significant concerns about the potential risks of COVID-19 vaccination, particularly regarding fetal health and their own well-being. This indicates a gap between perceived risks and actual vaccine safety data, emphasizing the need for better communication and reassurance regarding COVID-19 vaccination during pregnancy.

Negative social influences, such as perceptions shaped by social media and discouragement from family and peers, further reinforce reluctance. Social media plays a role in shaping perceptions, while opinions from family and peers can further discourage vaccination. In the Southeastern Asian region, Malaysian citizens are the largest internet users, with a reported internet penetration rate of 97.4% in 2024 [30]. As regards social media influence, it contributed to pregnant women’s hesitancy and refusal of the vaccine during pregnancy, as a lot of misinformation, such as the COVID-19 vaccine, caused harm being spread virtually [26]. Negative social influences from spouses, family members, and peers had a substantial impact on pregnant women’s willingness to accept the COVID-19 vaccine. Spouses and family members are the individuals closest to the pregnant women, and the most reachable advisors and trusted individuals which involve in their decision making [23,24,31]. Living in the rapidly developing technological era has given pregnant women better access to digital information sources regarding the COVID-19 vaccine. Nevertheless, they were at risk of exposure to misinformation and misconceptions about the COVID-19 vaccine for pregnancy.

Additionally, some pregnant women believe that life has returned to normal, reducing the urgency for vaccination. Malaysia has reported a marked reduction in the number of new cases of COVID-19 infection since the initiation of nationwide COVID-19 vaccine rollouts in February 2021 [32]. In addition, the COVID-19 tracker has stopped reporting the cases since 6 October 2022 [33]. In contrast to the pandemic era, news of COVID-19 hospitalizations or deaths was no longer broadcast in mass communications. MOH has also lifted the rules of wearing masks and social distancing in public areas since 19 December 2023 [34]. Consequently, pregnant women felt that the current situation had returned to normal, and the COVID-19 virus was not a threat anymore. The new norm phenomenon had affected pregnant women’s willingness toward COVID-19 vaccine as they perceived a low risk of contracting the infection [20,22,25]. The perception that life has returned to normal has contributed to reduced urgency for COVID-19 vaccination among pregnant women. The decline in reported cases, the cessation of COVID-19 tracking, and the lifting of public health restrictions have reinforced the belief that the virus is no longer a significant threat.

Vaccine skepticism and prejudice, driven by conspiracy theories and doubts about its effectiveness, also play a significant role in their decision-making. Conspiracy theory and the belief human beings were used as experimental subjects were the significant factors impeding pregnant women’s willingness to the COVID-19 vaccine during pregnancy [22,27]. The theme that emerged was “I am not going to be a guinea pig”, indicating pregnant women were unwilling to participate in COVID-19 vaccine trials [27]. Furthermore, pregnant women were easily exposed to conspiracy theories while actively navigating the internet and looking for vaccine-related information [31]. A mixed-method study revealed 91% rumors and 9% conspiracy theories from 637 COVID-19 vaccine-related online sources from 52 countries including rumored to be used as guinea pigs for vaccine trials [35]. In addition, being a novel vaccine that was produced in a short period without sufficient research has made pregnant women more hesitant [22,25] or reluctant [6]. In line with other studies, pregnant women were uncertain about the vaccine’s effectiveness for pregnancy [24,25] and believed vaccines were ineffective [26]. Vaccine skepticism and prejudice, fueled by conspiracy theories and doubts about effectiveness, significantly influence pregnant women’s hesitancy toward COVID-19 vaccination. The fear of being used as experimental subjects and exposure to misinformation online further reinforces their reluctance.

### Facilitators & implications for practice and policy

The facilitators for pregnant women’s acceptance of the COVID-19 vaccine are multifactorial. In our study, three main themes were identified including concerns about the effects of COVID-19 infection, trust in the vaccine and personal and shared vaccination experiences. When individuals are concerned about the risk of contracting COVID-19 or believe the disease can cause serious health consequences, they are more likely to perceive the threat as high as described in HBM (perceived susceptibility and perceived benefit) [11,13–17]. Having personal and shared experiences is reflecting a cue to action in HBM [11,13–17]. Having trust in the vaccine is a form acceptance attitude toward vaccine which illustrated in TPB as well as personal and shared experiences can shape subjective norms [12–15,18,19]. Emphasizing the risks of COVID-19 infection during pregnancy by health authorities, including potential complications for both mother and baby as well as integrating the discussion by healthcare providers during antenatal visit are crucial.

A key factor facilitates pregnant women’s acceptance of vaccine was their increased concern about the potential impact of illness on both their own health and that of their unborn babies [6,25,27]. Additionally, participants believed the COVID-19 vaccine could vertically provide their unborn babies with the antibodies for protection during infancy [36]. They therefore supported the effectiveness of the COVID-19 vaccine for both pregnant women and their unborn babies. Pregnant women who accepted the vaccine also believed the vaccine was safe for themselves and their unborn babies [6]. In our study, this belief emerged as a result of an uneventful previous COVID-19 vaccination experienced by the participants, either before or during pregnancy. Participants’ experiences of observing the effects of the COVID-19 vaccine in curbing the viral transmission effectively in their family members had also facilitate their acceptance. Family experiences served as impactful cues to action whenever participants observed that their relatives were less likely to contract COVID-19. It acted as a compelling prompt for them to consider vaccination significantly. Pregnant women felt more motivated to receive the COVID-19 vaccine after observing others especially family members, friends, other women, young adult, neighbours, and prominent people in the country getting vaccinated and recommending it [26,37,38]. Seeing positive experiences from people they trusted or others with similar backgrounds helped to reduce their fears and reinforced their confidence in the safety and benefits of the vaccine. This highlights the importance of social influence in shaping one’s attitude and action as regards vaccination acceptance.

Pregnant women viewed the COVID-19 vaccine through the maternal lens – they outweighed the COVID-19 vaccine benefits and risks on their unborn babies first before writing down their signatures on the consent form. They can also be at the stage of ambivalence, whereby they neither accept nor refuse the vaccine for many reasons. Vaccine hesitancy is a complex issue, particularly in a multiracial country like Malaysia, whereby the attitudes of pregnant women are influenced by personal, sociocultural, and institutional factors. HCPs play important roles in recognizing these pregnant women at what stage they are so that targeted counselling and vaccine education can be provided in a timely manner [22,23,39].

## Strengths and limitations

The study uses a hermeneutic phenomenological design with in-depth interviews. This allows for a rich understanding of the complex reasons behind vaccine hesitancy and acceptance among pregnant women, capturing nuances that quantitative studies might miss. The study specifically addresses pregnant women, a group at higher risk of complications from COVID-19. This focus is crucial for developing targeted interventions. By examining both barriers and facilitators, the study provides a balanced perspective, which is more helpful for designing effective interventions. However, there are several limitations for this study. It was conducted only in Kelantan, Malaysia, so the results may not be generalizable to pregnant women in other regions or countries with different cultural, social, or healthcare contexts. Participants’ recall of past vaccination experiences or social media information may be subject to recall bias, affecting the accuracy of the data. The sample primarily consisted of Malay pregnant women, limiting the generalizability of the findings to other ethnic or racial groups.

## Conclusions and recommendations

This study provides important insights into the complex interplay of barriers and facilitators that shape pregnant women decisions regarding COVID-19 vaccination. The barriers reflect both individual-level concerns and a social dynamic, including the impact of misinformation and distrust in health systems or authorities. Conversely, the facilitators often served to counterbalance hesitancy by reinforcing the perceived benefits of vaccination and reducing uncertainty. These findings underscore the need for multifaceted public health strategies that go beyond simply providing factual information. Efforts should focus on rebuilding trust in vaccines, addressing misinformation within social networks, and amplifying positive vaccine narratives within communities.

## Supporting information

S1 File Semi Structured Guide in Malay with English translation. (DOCX)

S2 File Themes of barriers and facilitators in Malay Language. (DOCX)
