# Supplementary material for: A qualitative study on barriers and facilitators of COVID-19 vaccination during pregnancy among pregnant women in Malaysia
Source: PLoS One. 2025 Aug 8;20(8):e0329895. doi: 10.1371/journal.pone.0329895 (PMC12333975; doi:10.1371/journal.pone.0329895)
Supplement: S1 File — (DOCX) [file pone.0329895.s001.docx]

Appendix: Semi structured guide (Malay version with English translation)

| Soalan (*Question*) | Pertanyaan lanjut (*Further questions)* |
| --- | --- |
| Isu spesifik:  Meneroka keraguan vaksin COVID-19 dalam kalangan wanita hamil.  *Specific issues:*  *Exploring COVID-19 vaccine hesitancy among pregnant women.* | |
| 1. Nyatakan pandangan anda berkenaan vaksinasi COVID-19 dalam kalangan wanita hamil?   *Express your views on COVID-19 vaccination among pregnant women?* | Anda mempunyai (ya/mungkin) keraguan terhadap vaksinasi COVID-19 semasa hamil, boleh kongsikan kenapa anda berpandangan begitu?  *You have (yes/maybe) doubts about COVID-19 vaccination during pregnancy, can you share why you have that view?*  Boleh terangkan sebab keraguan anda?  *Can you explain the reason for your doubts?*  Boleh anda jelaskan apakah yang menyebabkan anda meragui vaksin tersebut.  *Can you explain what makes you doubt the vaccine.*  Apakah bentuk keraguan anda terhadap vaksin?  *What is your type doubt that there is a vaccine?*  Pandangan keberkesanan vaksin COVID-19 semasa hamil.  *Views on the effectiveness of the COVID-19 vaccine during pregnancy.*  Pandangan keselamatan vaksin COVID-19 semasa hamil.  *Views on the safety of the COVID-19 vaccine during pregnancy.* |
| 1. Apakah yang mendorong anda meragui vaksin COVID-19?   *What makes you doubt the COVID-19 vaccine?* | Adakah ahli keluarga, kenalan, ahli masyarakat, kakitangan perubatan, media massa atau lain memainkan peranan dalam memberi dorongan kepada anda untuk anda berpendapat sedemikian?  *Did family members, acquaintances, community members, medical personnel, mass media or others play a role in encouraging you to have such an opinion?*  Adakah anda pernah mendapat maklumat yang meragukan berkenaan suntikan vaksin COVID-19 semasa hamil?  *Have you ever received dubious information regarding COVID-19 vaccination during pregnancy?*  Adakah anda mempercayai atau mempunyai keraguan terhadap berita yang diterima?  *Do you believe or have doubts about the news you receive?*  Bagaimana anda mengesahkan kesahihan maklumat yang diterima?  *How do you verify the validity of the information you receive?* |
| 5. Adakah pengalaman dan pendapat anda berkenaan keraguan suntikan vaksin COVID-19 memberi pengaruh kepada orang lain?  *Do your experiences and opinions regarding COVID-19 vaccine skepticism influence others?* | Apa pandangan orang lain apabila berhadapan dengan anda yang mempunyai keraguan terhadap suntikan vaksin COVID-19?  *What do other people think when faced with you who have doubts about the COVID-19 vaccine?* |
| 1. Pada pendapat anda yang mempunyai keraguan vaksin COVID-19, bagaimana keraguan vaksin semasa hamil dapat diatasi?   *In your opinion, those of you who have doubts about the COVID-19 vaccine, how can vaccine doubts during pregnancy be overcome?* | Pada pandangan anda, apakah cara-cara yang boleh megurangkan keraguan terhadap suntikan vaksin COVID-19 semasa hamil.  *In your opinion, what are the ways that can reduce doubts about getting the COVID-19 vaccine during pregnancy?* |
| Isu spesifik:  Penerimaan vaksin COVID-19 semasa hamil.  *Specific issue:*  *Receiving COVID-19 vaccine during pregnancy.* | |
| 1. Boleh berikan pandangan anda kenapa anda tidak sepenuhnya menerima vaksin COVID-19 semasa hamil?   *Can you give your opinion on why you did not fully receive the COVID-19 vaccine during pregnancy?* | Pada pandangan anda, sekiranya anda tidak menerima vaksin COVID-19 semasa hamil, jelaskan risiko yang bakal anda hadapi.  *In your opinion, if you do not receive the COVID-19 vaccine during pregnancy, explain the risks you will face.*  Apakah kebaikan jika menerima vaksin? Keburukan jika menerima vaksin?  *What are the advantages of receiving the vaccine? What are the disadvantages of receiving the vaccine?*  Apakah kebaikan jika tidak menerima vaksin? Keburukan jika tidak menerima vaksin?  *What are the advantages of not receiving the vaccine? What are the disadvantages of not receiving the vaccine?* |
