# Supplementary material for: A qualitative study on barriers and facilitators of COVID-19 vaccination during pregnancy among pregnant women in Malaysia
Source: PLoS One. 2025 Aug 8;20(8):e0329895. doi: 10.1371/journal.pone.0329895 (PMC12333975; doi:10.1371/journal.pone.0329895)
Supplement: S2 File — (DOCX) [file pone.0329895.s002.docx]

Themes of barriers and fasilitators

THEMES FOR FACILITATORS

| Themes | Subthemes |  |
| --- | --- | --- |
| 1. Personal and other people’s vaccination experience |  | P3 – **”Ambik (vaksin) tu takdok raso gapo.. okay.. badan pon okay la.. takdok gapo..”**  P5 – **”Dia jadi lepas ambik (vaksin) tu dia jadi efek kepada saya punya.. tidok kato kesihate yang teruk.. cumo dia raso sakit kepala.. tapi tak lamo pun.. tiga hari dia jadi sakit kepala.. tapi takyah risau la.. dia macam sekejap je macam tu.. saya tengok orang sebelum-sebelum ni takdok gapo pon.. dio ambik booster pon takdok gapo.”**  P6 – **“Sayo biaso vaksin maso mengandung dengan anok yang pertamo..**  Maso cocok vaksin hari tu 14 haribule 10.. yang keduo tu 14 haribule berapo tok ingat doh.. **Okay lah .. bagi sayo okay sangat-sangat vaksin ni .. takdok gapo la. dengan vaksin sayo raso sihat. Molek berdasarkan pengalaman vaksin”**  P7 – ”**Saya tanya lah yang dah experienced dah ambik kan yang first dose tu, saye tanya OK tak ? die cakap OK je takde ape takde masalah bagi die.**  **Tapi orang mengandung hari tu rasanye takde, takde dengar la ade masalah (dekat vaccination center).”**  P9 – “Tapi kalu ikutkan sebelum ni, ramai kato lepas (cucuk) vaksin menyebabkan sakit kae, tubuh lemoh**. Tapi setakat ni alhamdulillah lah bagi sayo, takdok gapo sebab sayo dah ambik duo dos. Maksudnyo takdok gapo masaaloh lah. Pengalaman (divaksin) biasa (OK) je**. First dose tu demam lah, sebab ubat dia kuat kut. Ha, demam, tapi dos kedua tu alhamdulillah takde apa-apa.”  P10 – “Saya masa vaksin first dose dengan second dose takdok side effect sangat pun.. biaso ja. Booster baru ada side effect, tu pun demam 2 3 hari jah. Lepas tu.. hok tu je lah**. Saya punya vaksin side effect takdok la sekuat macam ore duk kato .. takdok lah, saya tak raso semua tu. Alhamdulillah side effect saya sikit jah. Booster yang saya rasa.. lenguh-lenguh tangan pun tak sangat pun.”**  P11 – “Takdok efek (vaksin) gapo-gapo pun, OK ja. Maksudnya simptom-simptom biasa jah, penat awal-awal tu, macam simptom orang tak mengandung. Ambik vaksin (masa mengandung) tu, ada efek awal-awal tu pening kepala, makan panadol .. lepas saya ambik vaksin tu, doktor bagitau terus ambik panadol. So, OK lah, tu jelah. **Kalau saya sendiri pun memang saya rasa benda tu (vaksin) so far alhamdulillah lah, maksudnya kita lebih kuat antibodi daripada hok tak ambik vaksin. Baby saya pun keno covid jugok 2 bule lepas dio lahir, tapi dia takdok simptom sangat, dia deme sahaja. Saya rasa benda tu dibantu oleh vaksin saya ambik semaso mengandung.”**  P13 – “Saya pulak, sebagai orang mengandung, dalam masa 5 tahun saya baru sekali je kena (jangkitan covid).. alhamdulillah, risiko teruk tu takde. Cuma demam biasa-biasa je. **Untuk first dose and second dose memang saya tengah pregnant, takde apa-apa .. lepas saya dapat vaksin COVID19 semaso mengandung tu saya takde ape-apa efek lah.** Lenguh tu macam biase lah kan. Tapi demam ke ape ke, sesak nafas ke takde. Booster tu lepas bersalin lah. Tu je lah. Kesan dia biase je, takde ape-ape pun.”  P17 – **“Vaksin ni rasa takdok raso gapo-gapo. Takdok raso sakit ko dok lah. Takdok raso sakit, macam biasa. Mace takdok gapo-gapo. Mace takdok efek gapo-gapo lah. Takdok efek lah kepado diri sayo ni. Tapi takdok raso apo pun,** takdok efek pun. Takdok raso deme, takdok efek jantung. Ha, OK.”  P16 – **“Takdok gapo-gapo, takdok deme... Alhamdulillah lah, sihat.”**  P18 – **“Takdok raso gapo pun. OK jah. Takdok lah simptom pelik-pelik tu, takdok. Mace ore kato (menyebabkan) letih ko gapo takdok. OK jah, mace biaso jah. Saya ambik (vaksin) dos kedua kalau tak silap masa mengandung anak no. 2. Lepas tu OK jah. Maso gi cucuk tu sayo alahan gini lah .. tapi gi cucuk mase biaso... takdok gapo pun, pahtu balik rumoh pun OK jah... takdok efek gapo-gapo lah. OK. Takdok masalah. Saya ambik (vaksin) tu meme sayo takdok masalah dengan vaksin COVID19. Memang alhamdulillah, semuanya OK.”**  P19 – **“Masa vaksin dulu tu, kesan dio macam bengkak-bengkak gitu jelah tempat suntikan. Yang lain tu takdok lah, OK jah. Demam tu takdok lah. Semua OK lah. sebab kita pun dah penah lalui benda tu (vaksinasi)...** kito pun tokse penyakit ni berulang lagi... kalau boleh nak kurangkan lah risiko tu...  Sebab dah keno (jangkitan covid) sekali tu perit jugok la keno...”  **P10 – “Sebab adik saye ni askar, wife die pregnant, dia ado gi kursus, balik tu dio kena covid. kebetulan wife dia tu pregnant anak nombor dua, tapi wife dia dah ambik second dose dah lah, alhamdulillah wife dia tak dijangkiti (covid), OK lah, sampai kat dia jah. Tok berjangkit lah kat wife dia**, sebabnya dia deme tu tak kuarantin lagi, second day deme baru dia kuarantin. Kuarantin dalam rumah je. So saya rasa, **kalau pendapat saya, OK untuk ambik (vaksin) tu untuk prevention, untuk kurangkan sikit dia (jangkitan covid) punya simptom.”**  P10 – ” Vaksin ni mungkin atas imun ore jugok lah. Ibu dengan abah saya alhamdulillah ambik booster takdok masalah apa. So, saya rasa vaksin tu atas badan masing-masing jugok lah nok terimo vaksin tu macam mana. Mungkin ada mostly hok keno side effect tu jadi.. dah digembar-gembur, jadi orang lain pun takut kan, so jadi tak ambik. **Kalau ada yang pengalaman macam saya ni takda sangat side effect yang teruk-teruk, jadi saya ambik lah.”** |
| 1. **Trust in the vaccine** | 1-Protection from covid-19 infection | P1 – **”Ado sesetengoh oghe mace bilo vaksin ni ado antibodi kuat. Bulih elak penyakit.. jangkitan COVID19 ko.. Anok bulih dapat imuniti (perlindungan).”**  P2 – “Yang booster tu ramai yang kena tambah lagi.. tapi sebenarnya masa tu saya dah pun dapat virus covid.. saya dah kena covid.. **tapi bagi saya saya nak jugak ambik booster untuk tambah sebab saya dah kena (jangkitan COVID-19)... macam untuk tambah ketahanan saya lagi.”**  **P3 – “Untuk mengelakkan gotu, mace virus merebok, pahtu keno kuarantin tu jelah. Vaksin ni untuk mengelakkan jangkitan virus tu lah.. covid19. Sebab ambik vaksin tu kemungkinan takdok jangkite lah pahtu okay .. lepas daripado deme lenguh tu okay lah.. mace biaso jah.”**  **P4 – “Cegah anok daripada keno COVID19. Cegah ni lah x se wi anok keno covid jugak selepas lahir .. kae ado kae budok bayi hok keno covid lepah daripado lahir.. cocok vaksin x se wi.. cegah.”**  **P5** – **Menjaga diri saya sendiri.. dan untuk baby lah.. Penting la untuk mengandung sebab mencegah daripada keno jangkitan (COVID-19).** Ha imuniti.. mace mencegoh la.. dia tak kata kito takkan dapat COVID19 , cumo dio mencegoh.. kalu mungkin akan dapat cumo dio tak teruk mano.. dio mencegah daripada lebih teruk lagi.. penyakit ke..”  P7 – “Bagi saya bagus untuk ambik vaksin tu kalau kita takde rase ragu-ragu untuk ambik sebabnya at least die protect kite daripada kesan covid yang teruk. Macam saya cakap tadi lah untuk protection, **untuk protection memang bagus lah, at least protect kite daripada COVID19 tu. At least ade protection lah, untuk ibu, untuk anak sekali, maknenya macam kite jugak orang yang normal dah ade vaksin at least kalau kena COVID19 sekali pun takde lah benda tu harm atau makin worse.”**  P9 – “Kalu ore ngandung nak ambik pun, bulih ambik.. sebab nak elak daripada nyakit (jangkitan COVID19). Maksudnyo nak elak nyakit ni tokse wi, covid ni kae berjangkit. Kalau kita ambik vaksin ni mungkin kita boleh elak daripada berjangkit kepada kita sendiri lah. **Tapi sebab kito nok jago diri kito dari ore lain kae. Kito pun ado anok kecik, jadi ambik jugok lah. Tapi bilo ore ramai keno (jangkitan covid), sepupu pun ado keno, ha takleh ni, keno ambik (vaksin) sebab kito ado anok kecik, bahayo (jangkitan covid).”**  P11 – **“Protection untuk baby, kalau kese sampingan (vaksin) tu takda la rasa, saya rasa lebih kepada protection kepada baby sekali. Kuatkan baby jugok.”**  P13 – “Yang buat saya aware lah kan, vaksin ni dia satu macam pelindung. So, kalau kita.. memang dia bukan untuk baik (penyembuh), dan dia mungkin jugak ada kesan sampingan sebab dia (vaksin) benda yang baru kan, tapi **at least kita dapat kurangkan risiko kita untuk dapat penyakit itu (jangkitan covid) yang lebih teruk. Itu daripada fahaman saya lah, daripada awareness saya. Sebab tu saya ambik (vaksin). Yang saya tahu dia (vaksin) kurangkan risiko jangkitan covid. Dia tak efek kepada bayi.”**  P17 – **”Bagus untuk diri kito jugok. Elak jangkit dengan COVID19. Takdok sakit-sakit (jangkitan covid) dah.”**  P19 – ” Bagi saya vaksin ni baik lah untuk kesihatan kite. Walaupun tak boleh cover 100% tapi kita mengurangkan risiko jangkitan (merebak) kepada orang lain, anak-anak kita, mak ayah kita. Yang saya faham lah. Dengan ada vaksin ni boleh mengurangkan risiko kematian, mengurangkan risiko yang teruk. Tapi lepas kito ambik (vaksin) tu nok sembuh 100% tu dok, ado jugok (risiko) keno jangkitan covid tapi tak teruk sangat. Mace berair hidung gitu jah.”  **P10 – “Duo-duo samo jah. Kalau tak ambik vaksin, keno covid pun risau jugok. Kalau ambik vaksin pun, duo-dua berisiko. Cumonya kita punya mindset kena ambik vaksin (supaya) dia (jangkitan covid) punya simptom tu kurang. ~~Covid still boleh kena, Cuma tempoh simptom tu boleh kurangkan.~~** **~~Adik saya hok bongsu keno covid sebelum vaksin, dia hilang deria bau selama 2 bulan jugok, lepas tu second time dia kena (jangkitan covid) selepas cucuk vaksin dia punya simptom ringan sikit. Simptom tu 3-5 hari je.”~~**  P18 – **”Cuma saya fikir untuk kesihate untuk lindung diri dari COVID19 gapo tu. Ye lah, kalau kito ambik vaksin tu mungkin kito kuat sikit daripado dijangkiti COVID19 kae. untuk anok jugok.”**  P19 – **”Risiko (jangkitan covid) tu kurang la berbanding tak ambik vaksin ni. Jangkitan tu ado tapi tok teruk sangat. Tapi kurang risiko nok gi ke teruk tu.”** |
|  | 2-Perceived safety | P3 **– “Raso selamat… Sebab lepas beranok tu saya cocok tak dok masaloh gapo pun .. mungkin bagus kut untuk ibu mengandung.. raso gitu lah.** Baby..pun okay..  **”**  P6 – “Sayo raso molek lagi untuk ore ngandung.  **Keselamate.. takdok gapo.. raso okay jah.. raso selamat ambik vaksin.”**  P12 – “Pada pendapat saya lah kan, kalau benda (lemak khinzir) tu .. kerajaan kita ataupun majlis agama kita pun bagi kebenaran untuk orang Islam lah untuk ambik vaksin, saye rasa takde masaalah, takde keraguan bagi diri saye. Ade DNA babi ke, ape ke dalam tu. **Rasanya dia (vaksin) dah selamat bile KKM dengan majlis ulamak sahkan memang vaksin ni selamat untuk diri kite, saya rasa takde masalah untuk ambik.** Tapi pendapat setiap individu kan tak same. **Sebab saya yakin ape yang kerajaan dah sarankan dan majlis agama bila diorang dah keluarkan fatwa yang mengatakan vaksin ni kan hukumnya harus.”**  P14 – “Pendapat saya, saya fikir gini, tak kan lah.. benda tu untuk kebaikan kito. Pastu takkan lah ore hok nok bagi ko kito tu tak consider kito ni agamo gapo semo. Jadi, dio lagi lah berilmu daripada kito hok dapat ni. **Mesti dio consider belako. So, mace tok was-was lah, sebab dio mesti buat kajian dulu sebelum bagi kat kito. Saya tok raso pun ado babi dale tu (vaksin). Sebab saya yakin vaksin ni selamat lah.”** |
| 1. Concerns on the effects of COVID-19 infection | 1-Perceived susceptibility | **P2 – ”Tapi saya rasa macam saya orang kesihatan yang mengandung saya mudah terdedah dengan apa-apa (penyakit) sahaja.. so saya ambik booster untuk (lindung) badan saya jugak..”**  **P5 – ”Sebab saya mengandung kena jage jugok keselamatan anok saya dalam kandungan ..** **jadi kalau saya kena covid, anak saya pun akan keno efek (COVID-19) lah.. same macam saye..”**  P7 – **“Tapi saya as health worker memang saya ambik vaksin tu, saya tak kesah lah, untuk protect saya sendiri sebabnye wad saya sendiri lepas tu dijadikan wad COVID19.** Macam kitorang yang healthcare worker ni jadi macam terpaksa ambik jugak sebab kite yang akan berhadapan dengan ni lah (virus COVID19) .. kadang-kadang kite tak tahu kan yang masuk wad tu, yang masuk check check positif COVID19 dalam wad kan, kadang-kadang yang duduk dalam wad pun sendiri ada yang positif lepas tu, padahal duduk dalam wad jugak, ha jadi bagi protection untuk kite la. Perlindungan untuk kite. **Macam kite healthcare worker ni terdedah selalu dengan pesakit jadi kite ade kita punya kesedaran sendiri.”**  **P8 – ”Untuk mencegah COVID19 kepada ibu mengandung mungkin kalau tak mencegah pun, nak mengurangkan lah risiko untuk kena COVID19 (yang teruk) tu.. sebab ibu mengandung ni mudah terkena penyakit.”**  **P9 – ”Ibu mengandung ni bukan sihat macam orang sihat. Antibodi pun tak kuat mana. Kita ni kan dua orang, kena fikir kandungan kite jugok.” Nak kato risau banyok tu dok jugak, tapi lebih risau kepada jangkitan covid. Sebab contoh, dalam ahli keluarga sore keno covid, pahtu nyaknyo lah ore lain. Anak-anak kita kecik lagi. Risau jugok kalau keno covid. Maksudnyo tokleh nok jupo dengan puok dio (ahli keluarga).”**  **P10 –”Even lo ni pun, hok takdok covid pun jangkitan semua pun mudah dapat kat ibu mengandung. Kalau covid, lagi teruk dio punya risiko (Jangkitan). Selesema dengan batuk pun mudah berjangkit lo ni,”**  **P16 – ”Saya raso antibodi ore tok ngandung ni dio kuat sikit, orang mengandung ni dio sebab dio bawak duo (badan) kae... mungkin kure sikit lah imunisasi tubuh. Mace cepat terdedah kepada deme, selesema.”**  P19 – **”Imuniti (orang mengandung) dio rendah sikit kan setakat yang saya tahu... kalau kito tok mengandung tu lain lah sikit...”** |
|  | 2-Perceived severity | **P1 – ”COVID19 kae kade2 dio bulih bowok ko kematiae, bulih penyakit kronik**.. jadi mace rungsing la.. **kalu kito ngandung kae.. keno jangkitan COVID19 koho bahayo**. **Dio bulih menyebabkae.. bulih buwi kese la kepado anok.. penyakit ko.. paru-paru anok ni koho ado penyakit kronik ko..** gano ko.. tu jelah.. jangkitan kume ko..”  **P5** – **Mungkin bersalin awal (kalau keno covid).. kalau awal sangat macam tak cukup bulan tu tak boleh survive. lepas tu kalau macam kena COVID19 mase mengandung ni.. macam artis tu siti sarah tu dia meninggal masa mengandung.. ha takut macam tu la.. die efek kepada kandungan.. takut meninggal mase bersalin.. sebelum bersalin.. takut kita masa awal-awal kandungan ni.. kalau kita tak ambik.. mungkin jadi efek la.. memang kena ambik vaksin la..**  P6 – **”Jadi hidup pun takkan lamo bilo keno covid. Kalu mok dio, kemungkinan gitu jugok lah kalu (keno) covid .. mace mudoh.. hidup tok lamo lah. bulih meninggal.** Maso mengandung ni macam... saya raso mace tu jugok lah. **Mungkin bile lahir awal bayi tu tak sempurna, so bimbang ke arah tu lah, ataupun die ade kena kanser gapo-gapo ko.”**  P7 – **“Sebab saya pregnant ni umur dah 42 .. mungkin kena ambik (vaksin), kalau saya tak ambik langkah-langkah pencegahan lain, saya takut makin worsening dekat saye.. banding dengan umur sekarang lepas tu pregnant pulak** at least kalau ade something yg dah di-offer tu boleh dah nak ambik.”  P8 – ”**Cumo kalau lah COVID19 semaso mengandung rasonyo mace bahaye lah. Vaksin dengan covid, keno covid lah rase bahaye.** Keguguran atau terberanak awal lah. **”**  P10 – **”Saya rasa komplikasi covid meme.. tinggi risiko kalau keno (jangkitan covid) tu. Sebab kalau kat HRPZ pun penuh dengan ibu mengandung dan baby jugok kan, hok keno covid. kalau covid tinggi lagi risiko dio. Ibu tu mungkin macam ada radang paru-paru ke, kebanyakan saya dengar efek dekat ibu jelah.”**  P10 – ”Dia vaksin COVID19 untuk ore ngandung ni, viralnya Siti Sarah kae, hok hari tu semua orang pun tahu Arwah Siti Sarah tak sempat ambik vaksin lagi dok, **maso tu mostly hok pregnant ni ambik (vaksin) sebab dia tau kalau tak ambik lagi teruk dia (jangkitan covid) punya efek kan.** Masa tu saya takdok kat KKM lagi, tak pasti sangat die punya cerita sebenar. So saya rasa, kalau pendapat saya, OK untuk ambik (vaksin) tu untuk prevention, untuk kurangkan sikit dia (jangkitan covid) punya simptom.” |

THEMES FOR BARRIERS

| Themes | Subthemes |  |
| --- | --- | --- |
| 1. Fear of the side effects | 1-Previous unpleasant side effects | P1 – “Sayo kae ado doh pengalame ambik dos 1 ambik dos 2.. antibodi tu mace jadi lemoh.. takut ambik booster ni takut koho tu koho teruk lah. Sebab bagi saya lah sebelum saya ambik vaksin apo sebelum ni, sayo jenih nok deme ko, nok sakit ko payoh.. jadi bilo ambik vaksin ni jadi mace jadi lemoh.. tubuh bade jadi lemoh.. cepat deme tokleh keno uje sikit deme laluh.. kalu deme pon yo mace.. kalu sebelum vaksin ni sayo deme pon sehari ko duo hari ko mace ile selalu.. tapi bilo ambik vaksin ni mace seminggu tok bangun.. mace koho teruk laa.. daripado napoknyo..  P2 – “Sebab bila saya kena booster tu saya macam terkena balik macam simptom covid balik.. saya jadi macam tak rasa.. sedangkan saya baru je lepas habis covid dalam sebulan.. saya start demam badan saya lenguh so saya rasa untuk kali keempat (booster) mungkin saya tak ambik kut.. sebab saya terasa booster tu dah teruk untuk saya..”  P5 – “Dia jadi lepas ambik tu dia jadi efek kepada saya punya (badan).. tidok kato kesihate yang teruk.. cumo dia raso sakit kepala.. tapi tak lamo pun.. tiga hari dia jadi sakit kepala.. sakit kepala berdenyut.. lepas tu macam penat.”  P14 – “First, dia punya side effect la kae, saya rasa lah lepas saya cucuk tu macam makin .. nak royak lemah tak lemoh.. tapi pada saya, saya rasa alergik gitu. Nak royak gano eh.. cepat gatal-gatal. Tapi tahu-tahu nyo, lepas cucuk tu, mudah gatal-gatal, raso mace badan gatal-gatal gitu. Lagi, mengah tu jelah. Demam biasa lah kan. First tu, lepas cucuk vaksin terus deme. Second dose tok deme. Hok third dose tu lepas cucuk tu terus raso kesan dio. Kese sampingan tu pening, deme 2 hari. "  P15 – “Sebab saya ingat saya cucuk vaksin dulu meme ado deme la jugok.. deme, sakit kepalo. Bilo dos kedua tu, sebab dio kuat sikit kae, maso tu jadi deme, sakit urat gapo. Mungkin lah hormon berubah sebab bendo baru (vaksin) masuk dalam badan.” |
|  | 2-Concern about harm to mother and baby | P1 – “Raso mace ibu mengandung ambik vaksin ni tak perlu kut sebab yo mace.. bagi efek la.. **bagi efek la ko ibu mengandung** .. sebab tok tahu lah.. lagi-lagi first mengandung kae..anok pertamo ni..tok tahu lagu mano antibodi kito. sayo tok ambik kut.. sebab supo sayo royak tadi.. **takut risau lah keselamate anok kae.. takut jadi gapo ko anok ko.. pahtu keselamate diri kito**..”   - Risau kepada keselamatan diri sendiri & anak   P4 – “Raso takut jugok nok ambik.. sebab kure percayo sangat kepado vaksin.. ha **takut efek kepado kemudian hari**. Ibu jadi mudoh deme, mudoh ado penyakit. Mudoh deme, sebelum ni payoh nok deme kae.. lepas pado vaksin tu jadi mudoh nok bulih deme... mace tak cekak.. mace po tubuh jadi koho letihhh.”   - Risau kepada diri sendiri – sebab: kurang percaya kepada keselamatan vaksin   P8 – “Vaksin COVID19 untuk ore ngandung eh.. bagi sayo vaksin COVID19 untuk ore ngandung mace agak **berisiko** sikit lah kut. Sebab ho lah vaksin kae..sebab kito pun ade kes ore hok vaksin ngandung .. lepas tu die ade, gapo ore panggil tu.. kese sampingan .. ha jadi mace kito tengok daripada kes siti sarah lah.. die mengandung lepas tu die meninggal.. tapi time siti sarah tu die vaksin dah ke? Tak tahu lah pulak, tapi bagi saye die (vaksin) berisiko sikit lah bagi ore ngandung.”   - Rasa vaksin ni berisiko – boleh bagi risiko kepada ibu mengandung   P11 – “Ore kato untuk jago, sebab takdok ubat lain lagi untuk protect dari jangkitan covid. Sebab tu lah 50-50, 50% tu yakin boleh protect, 50% lagi takut jugok, ado kese sampingan ko, especially saya tengah mengandung. Sebab takdok lagi (vaksin) sebelum tu kan, **vaksin COVID19 benda baru**. Setakat hok saya tahu, paling worse adalah gugur je la untuk ore ngandung.”   - Risau kepada keselamatan diri sendiri – sebab: vaksin ni benda baru   P15 – “Kalau mengandung ni kito fikir mungkin ado efek ko baby .. fikir ko kesihate kito.. kebanyakan mesti akan mikir mace **efek ke baby la hok paling risau** nyo. Kito pun tok tahu dale vaksin tu ado gapo.. kito pun tok tahu jugok penerimaan bendo baru dalam tubuh kito gapo semo. Jadi nyo risau sebab tu, raso kito pon tok tahu keadaan dalam kandungan kito pado maso keno vaksin tu. Sayo tak menolak pun bendo tu (vaksin), mungkin ado kebaikan dio jugok, tapi akan ado efek lah. Kito lagi lah tok tahu kepado baby ni boleh jadi.. contohlah, **kecacatan** ko gapo.”   - Risau kepada keselamatan diri sendiri & anak – sebab: takut kecacatan   P15 – “Kalau saya sendiri lah, sekarang ni sebab ngandung. Saya sebenarnya gini, saya ada lah sikit masalah kesihatan.. yang melibatkan hati, fungsi hati sayo tok molek, jadi doktor meme pesan doh panadol pun sayo tokleh make lo ni .. sebab hormon maso mengandung ni dio lain.. kalau saya makan panadol, fungsi hati sayo makin teruk. Jadi andai kata vaksin ni sayo keno ambik lo ni, mungkin sayo tolak jugok la kut, **takut atas kesihatan sayo**. Mace ibu ni, kalau kito sakit, kito takpo, kalau bulih tokse anok sakit.”   - Risau kepada diri sendiri – sebab: ada masalah kesihatan   P16 – ”Takut keno penyakit ko lepas cucuk vaksin. Mace mudoh keno penyakit ko.”   - Risau kepada diri sendiri   P12 – “Sebab sebelum ni pun penah ambik vaksin COVID19 saya rasa macam penat-penat badan**, kalau boleh taknak lah efek dekat bayi kite. Taknak lah ada risiko kese sampingan ke ape**, ha die macam tu. Lepas first dose kita cucuk rasa macam lenguh-lenguh badan, lepas tu kita punya tenaga macam kurang sikit.   - Risau kepada keselematan anak   P1 – ”Kese kepado ibu**.. takut ngandung bulih jadi kegugurae ko**.. jadi apo nih.. jadi alergik kepado belako ko.”   - Risau kepada keselematan anak - keguguran   P1 – “Anok ni kalu kito ambik vaksin mace jadi kure.. apo ni oghe kato.. gano eh nok royak.. jadi mace.. **takut jadi mace ado kekurange fizikal ko**.. **terencat ko gapo** ko kae.. nok bulih menyebabkae penyakit ko lepah beranok ko. Anok lah.. sayo risau kepada (kesihatan) kandungae.. keadaan baby lah.. kesihate ko, jantung dio.. takut mace tadi lah.. penyakit ko.”   - Risau kepada keselematan anak – kecacatan fizikal, mental retardation, heart problem   P2 – “Vaksin saya takut jugak .. kalau ambik vaksin saya takut ade kesan sampingan untuk baby saya sebab saya pun dah ade masalah. Saya dah ade pengalaman abort.. yang tu pun saya dah takut.. pastu macam-macam lah saya fikir.. tambah saya orang kesihatan.. **kecacatan baby.. baby saya premature**.. macam lah yang saya fikir doktor.”   - Risau kepada keselematan anak – kecatatan fizikal, lahir pramatang   P3 – “Sebab takut efek ke baby .. **takut kandunge lemoh lagu tu..** Takut terjejas untuk bayi raso gitu lah .. Ya takut nok ambik.. maso ngandung tu takut nok ambik vaksin.. takut ko baby lah. Takut baby ni kure aktif, pahtu gerok jantung dio tok gerok, haa rusing gitu.”   - Risau kepada keselematan anak – kurang gerak   **P4 –** “Sebab ni masalah dio takut ado masalah kandungae la**, takut gugur ko**.. Efek (vaksin) kepado bayi .. **bayi tok cukup umur.. beranok tok cukup umur.. ataupun kegugurae.. Raso mace po takut.. takut jugok la nok ambik tu..** takut kandunge masaaloh ko.. kalu kito ambik.. mace po.. apo nih.. kalu ambik vaksin takut jejas ko anok dale kandunge..”   - Risau kepada keselematan anak – keguguran, lahir pramatang   P7 – “M**ungkin first trimester kite ade alahan lagi, kalau nak ambik vaksin mungkin takut dengan ape-ape side effect selepas ambik, ha kalau bagi saye first trimester tu mungkin kalau saye sendiri pun akan ragu-ragu nak ambik.** Satu lagi takut **mengganggu perkembangan anak**, ha takut deformity hasil daripada vaccination tu sendiri.”   - Risau kepada keselematan anak – ganggu perkembangan   P8 – “Untuk saya, saya OK je, tapi untuk bayi tu, saya bimbang kepada keselamatan bayi lah. **Sama ada dalam perut, atau pun selepas beranak**. Kalau lepas beranak, faktor kesihatan die, mungkin lemah, sakit ke, kena duduk hospital lame ke.. lepas tu kalau dalam perut perkembangan die tak berkembang lah, tumbesaran tu terbantut ke apa semua.. ho saya bimbang kot tu jelah. **kalau bila saya ngandung ado baby tokse ambik.**”   - Risau kepada keselematan anak – penyakit kronik, ganggu perkembangan   P9 – “Macam ragu-ragu pun ada jugak, **takut baby lahir tak cukup bulan**. Takut nanti bilo dah tubik, macam-macam masaaloh ke baby pulok. Kalau tak mengandung OK je, tapi bila mengandung ni jadi mace.. sebab kite mengandung kae, takut efek ke baby jugok lah salah satunya.”   - Risau kepada keselematan anak – lahir pramatang, penyakit kronik   P12 – “Kalau mengandung ni tak sesuai sangat lah. Sebab ade efek dekat bayi sekali kan.”   - Risau kepada keselematan anak   P14 – “Bagi pihak saya lah, saya rasa saya tak ambik vaksin, bukan tak percayakan vaksin tapi.. untuk pregnant ni saya jadi ragu.. Cuma, saya rasa tak perlu kut waktu pregnant ni. **Mungkin kalau lepas tu (bersalin), boleh consider lagi lah**. Sebab saya takut bahan (vaksin) ganggu kandungan, even paracetamol kita tahu benda tu boleh je kae untuk pregnant, tapi saya takut-takut jugok. Vaksin.. lagi saya takut, sebab benda tu kito tok napok duk dale badan kito, masuk dale salur daroh gapo semo, dia boleh lalu ke baby apo semo, so meme takut lah. Lebih kepada takut, menyebabkan gangguan kepada baby. Takut ada efek kepada baby, contoh lah.. **cacat ko, atau mace baby tu ado masalah paru-paru ko**, masalah hok boleh diconsider lah. Kito tok tahu apa yang akan terjadi.”   - Risau kepada keselematan anak – kecatatan fizikal, penyakit kronik paru-paru   P16 – ” Tu lah, raso mace tok berapo nok yakin tu, takut mace ado efek ko baby ko, ha gitu lah. Mace bilo dah pregnant ni, sayo raso mace takut efek ko baby lah pulok dih.”   - Risau kepada keselematan anak |
| 1. **Vaccine skepticism and prejudice** | 1-Vaccine inefficacy | P11 – ”Saya punya keyakinan waktu tu untuk vaksin COVID-19 50-50 tu sebabnya saya akan baca lah, tengok sejauh mana kebersanan vaksin ni sebab kita punya vaksin tu.. waktu tu covid baru berlaku.. dan tak pernah berlaku pun covid19 sebelum ni, vaksin COVID19 pulak tak digunakan lagi, macam Pfizer, Sinovac, Astrazeneca tak pernah digunakan lagi sebelum ni, tak tahu pun benda tu boleh kurangkan (jangkitan) ataupun dok. Boleh betul-betul elakkan ke tak.”  P15 – “Dale kito cucuk vaksin pun saya masih terkeno jugok lah COVID19. Jadi sayo raso mace.. tok tahu lah.. nok royak pendapat gano.. mace tok pasti berkesan sangat pun, mungkin lah. . Yang dos pertamo dengan keduo tu, so bilo keno (cucuk) dos kali keduo tu dia mace berkurang (berkesan) sikit. Dio mace berkurang sikit, mungkinlah dio baru jugok la vaksin tu.. mace singkatkan maso (tempoh) jangkitan tu saya tak sure lah jugok betul ke dok.” |
|  | 2-Conspiracy theory | P2 – ”Saya rasa kerajaan ni buat eksperimen kat orang kesihatan, yang vaksin pun orang kesihatan yang kena ambik dulu..”  P8 – “Hok ni daripada apa yang saya baca lah, vaksin ni daripada tempat hok kene COVID19 jugok kae, betul dok? Wuhan.. ke mano? Jadi mace kito dok tahu gapo bendo dio letok dale tu.. bakpo die buat vaksin tu, bakpo tubik COVID19 tu.. so mesti la ade kemungkinan ni semua dirancang . Faedah vaksin untuk ore ngandung..hm.. bagi sayo takdok kut..sebab mungkin kerajaan ni nok bagi je kat orang mengandung tapi tak tahu die punye kese sampingan lain .. maybe kerajaan nok bagi ni nok jadikan tikus makmal je.”  P16 – “Ore tuo-tuo lah dih, kato vaksin ni nak ujikaji kito lah.”  P5 – “Sebab dia (suami) takut .. dia bukan takut dengan ubat, dia takut sebab orang ramai kate vaksin macam ada satu cip .. dimasukkan cip dalam tubuh kita.. mulo-mulo orang kampung kan.”  P4 – “Rasonyo samo lah.. sesetengoh ore kato ado dio masuk barang terlarang (cip) lah. Mace vaksin tu daripada mano tok tahu..”  P15 – ”Mindset dio mace takut lah bendo tu (vaksin). Dulu-dulu pun ado viral pasal vaksin ni ado cip lah gapo. Mace tu lah. Sayo pun tak sure sapo yang viral tu, tapi ada la kito terbaco dale satu facebook tu dio royak gitu, ado nok .. masuk cip dale tubuh kito tu .. jadi mace menakut-nakut lah ore untuk mengambil, sedangkan mungkin ado kebaikan. Maso tu meme ado lah viral pasal tu, tapi sayo tok ingat daripado mano bendo tu (sumber). Maso tu maso kerajaan galakkan ambik vaksin, lepas tu ado la tubik mace-mace berito untuk mempengaruhi ore lain.“ |
| 1. **Negative social influences** | 1-Social media’s shaping of perceptions | P3 – **“Mati sebab cocok vaksin hok beso dengar. Hok tu tengok dale telepon ..tiktok...”**  P4 – **“Takut anok dale kandunge lah masaaloh sebab banyok tengok dale facebook gapo kae kato efek daripado vaksin anok jaadi gini gini haa..** Gano eh.. jadi pesakit lah .. mace po mudoh sakit.. mudoh deme..Mudoh jadi sakit, mudoh deme.. Mace po .. anok tok sihat la.. **anok kure sihat.. beranok pramatang. Dale kandungae, mace po .. efek mace lambat.. mace tumbesare dio lambat.. bayi boleh meninggal.”**  P8 – **“Imun dia (bayi) akan jadi lemoh, jadi bayi tu cepat demam, cepat sakit, orang kate kalau sebulan tu mesti banyak kali gi klinik sebab die sakit, deme lah gapo lah, mutoh la, sawan lah. Dengar pun daripada dalam media sosial lah.”**  P11 – **”Sebab saya dah baca, bila saya masuk forum (media sosial) ibu-ibu mengandung ada bagitau bila dah vaksin dia gugur.. di awal pregnancy. Ha, baca benda tu. Di awal pregnancy ni dia high risk sikit kae.** Saya bila pregnant, memang ikut forum ibu mengandung dekat apps ibu mengandung. Dekat apps tu macam-macam ada, semua ibu mengandung share mace-mace dale tu. Semua info-info pasal pregnancy. So, adalah hok share pasal dia mengandung lepas vaksin dia gugur. Benda tu jugak lah yang buat kita 50-50 (ragu-ragu) tu. Takut lah jugok.”  P12 – **“Vaksin .. kalau yang saya ada bace dekat facebook, kalau Sinovac ni menyebabkan gastrik, lepas tu kalau sape yang ambik Pfizer jadi sakit kepala.”**  P18 – **“(Dari media social) Cakap boleh menyebabkan lumpuh lah, penyakit... padahal sebelum ni tak penah buat saringan perubatan pun.** Tak baik untuk kesihatan, macam saya cakap tadi akan menyebabkan lumpuh.”  P19 – ”Kalau kat facebook tu biasalah, ada yang palsu ada yang betul... kena pilih lah.” |
|  | 2-Influence from the family and peers | P4 – **”Dio (ore sekeliling) royak mace po kalu cocok vaksin ni banyok masalah la, dapat penyakit mungkin koho.. ore kato bukan koho baik jadi koho buruk.. jadi kesihate buruk la.** **Royak takyah cocok ni lah takyah cocok vaksin.”**  P4 – **“Husband.. kalu ngandung dio tok buwi lah.. Halangan.. vaksin sor.. pahtu suami tok bagi.. famili tak bagi.. gitulah. Saim pun ado jugak, dio royak tok payah ambik vaksin lah. Sebab masalah lah masalah kepado baby, kepada diri sendiri.”**  P7 – “Cakap-cakap orang lah.. cakap-cakap orang ooh kalau ambik vaksin ni jadi gini gini .. jadi makin memudharatkan, gitulah doktor. Omok-omokan orang jelah. Kalau ambik vaksin lagi teruk kite kena.. kita tak tahu benda ape yang disuntik kat kite.”  P6 – **“Saim.. keluargo sendiri.. royak takut jadi bendo lain pulok kalu ambik vaksin.. Raso bahayo Kalu mung ambik vaksin.”**  **P9 – “Tok tahu lah, sayo pun tok tahu jugok. Sebab dengar dari mulut ore tu kae. Salah satu yang saya dengar (dari ore sekeliling), lepas cucuk vaksin ni sakit. Antibodi tak kuat.”**  P10 – “Mungkin ada mostly hok keno side effect tu jadi.. dah digembar-gembur, jadi orang lain pun takut kan, so jadi tak ambik. **Macam contoh kat kampung abah saya tu dia (orang kampung) cucuk vaksin booster, lepas tu dia strok.. so, satu kampung dia tu tak ambik dah lah booster. Stop kat second dose je.”**  P11 – “**Mak bapak mertua saya tu macam konservatif sikit, so dia banyak takut lah. Lebih banyok takut daripado berani nak ambik vaksin ni. Banyak baca-baca kata negatif efek vaksin ni.** So bila nak ambik vaksin tu, dia orang ambik jugok, tapi keadaan hok mace .. **diorang (mertua) selalu share benda-benda negatif pasal vaksin.”**  P13 – “Pengaruh kut, daripada keluarga kut. Kalau keluarga dia terima benda tu, mungkin dia akan ambik. Tapi kalau pengaruh keluarga dia suka mendengar benda yang buruk, mungkin dia taknak ambik. Pengaruh sekeliling dia lah kut.”  P14 – **“Kalau saya sendiri diberi pilihan untuk ambik vaksin semaso mengandung ni, saya tanya orang sekeliling saya lah selain daripada diri saya.** Sebab penting jugak lah pendapat orang lain. Tapi saya memang takkan ambik. Contoh la kalau husband saya kata ambik, sebab tengok orang lain ambik (vaksin) takdok gapo-gapo, mungkin saya boleh consider tapi tak 100%. “  P17 – “Mulo-mulo ore lain cakap macam keno penyakit apo lah. Dengar ore royak kalau ambik pun tak molek jugok. Mace boleh sakit... ado benda-benda tak molek lah. Baik toksoh ambik.”  P18 – “Pelik tu mace ore royak, tak takut ko ambik vaksin maso mengandung... takut nanti jadi gapo-gapo ko anok ko... takut jadi gapo-gapo lah, takut ado efek. Dari jiran-jiran tepi rumoh sayo. Ado yang ambik dos ketigo tu ado yang kato tok berapo sihat, mudoh deme lah. Mudoh bulih penyakit lah.  **Lepas tu, suami saya pon tok galakkan untuk ambik dos ketiga (booster).”** |
| 1. Returning to normalcy |  | P2 – “Sekarang ni dah macam.. masalah perkara biase.. sebab kurang kite dengar orang ade gejala covid.. pastu yang kena covid pulak takde simptom.. sekarang kalau sakit mata pun hmm boleh jadi covid positif jugak.. hmm.. kalau kita diarrhea pun.. boleh jadi kena covid positif jugak.. so, macam sekarang saya tengok orang dah biase.. Ha.. tak sekuat seteruk dulu lah sampai ramai orang kena.”  P2 – “Ada yang dapat, cuma simptom die tak seteruk macam sebelum ni kite dengar sebab ramai orang dah ambik vaksin.. hm.. biasa mungkin yang teruk tu kalau takde vaksin lah kut.. sebab yang dah complete vaksin kebanyakannye simptom die biase je.. sakit mate.. diarrhea.. hm pastu macam sakit tekok.. kering.. macam batuk je.. so macam tak seteruk dulu.. sampai orang kena admit sampai CAT 4 CAT 5.. sekarang macam CAT 2 sahaja kalau ade pun.”  P5 – ”Saya mungkin dok la kut, sebab COVID19 pun dah berkurang, kalau dulu lagi teruk.. mungkin saya ambik kut kalau keadaan teruk, kalau sekarang mungkin saya tak perlu.. Sebab saya rasa saya dah ada dua dos kan, jadi cukup lah, tak perlu lah.. melainkan keadaan lagi teruk, macam COVID19 ni ada banyak kes meningkatkan, mungkin ada keperluan lah, sekarang takde dengar lagi kes pun, mungkin tak perlu kut.”  P7 – “Tak tahu lah doktor, sebab hari tu booster pun ade ramai je tak ambik, bukan yang mengandung lah, bagi diorang dah habis dah, musim COVID19 dah habis, kenape nak ambik booster lagi..  Cuma orang mengandung ni tak pasti lah apa tanggapan diorang.. macam fikiran orang lain lah jugak kut, sebab COVID19 tak banyak, dah takde sekarang kan, makin kurang makin kurang makin kurang mungkin diorang tak nak booster, cukup lah dengan first dan second dose je..  Ye lah, contoh sesetengah orang cakap “dah nak buat ape lagi nak ambik benda tu dah takde dah..” cukup cukup jelah dengan 2 dos tu, ramai je yang taknak ambik booster.”  P10 – “Sebab lo ni covid dah takdok sangat doh, saya rasa kalau lo ni pun jangkitan covid pun takdok mano doh, kalau tak ambik (vaksin) pun OK sebenarnya.. sebab kita pun ada influenza, even mengandung pun kena ambik ATT kan, ada vaksin jugok. Pastu, kalau maso outbreak dulu, better ambik lah saya rasa. Kalau lo ni kalau tak ambik (vaksin COVID19) pun OK jah, sebab takdok sangat dah COVID.”  P11 – “Bagi saya sekarang ni.. COVID19 sekarang dah jadi macam satu influenza, dah jadi macam satu jangkitan selesema, mungkin tak tahulah sebab apa. Manusia sekarang ni dah ada antibodi ko, setakat jadi simptom selesema, jadi batuk-batuk. Ubat hok diperlukan sajalah, even kalau kita keno covid sekarang ni pun, jumpa doktor macam dulu makan ubat, kalau batuk makan ubat batuk, banyak makan vitamin C biar badan tu lawan sendiri.”  P12 – “Lagi pun sekarang ni kan bila jangkitan covid dah kurang, jadi kite tak ambik port (tahu) sangat benda tu (pasal vaksin).. saya sendiri pun dah tak ingat ape yang berlaku, same macam awal-awal ada COVID19 dulu kan kita memang kalut nak cari apa rawatan terbaik untuk elak benda ni supaya tak kena dekat kite.”  P13 – “Sekarang ni, sebab semua orang mostly dah ambik (vaksin) so COVID19 tu dah macam takde lah teruk macam awal-awal dulu yang mana kita takde ambik (vaksin) langsung kan. So saya rasa mungkin penyakit lain pulak kut yang kita perlu risau.”  P14 – “Situasi lo ni biasa dah kut, sebab kita dah biasa kan. Takdok gapo doh la. Back to normal. Tapi kito tahu dia (jangkitan covid) ado lagi. Cumanya kita continue jelah life macam biasa.”  P15 – “Sebab lo ni mace kito anggap COVID19 ni mace deme biaso. Mace ore anggap COVID19 ni mace deme biaso jah, jadi kalau hok keno pon mace tak teruk sangat lah, tak mace dulu lah. Mace make ubat panadol gapo, pastu sembuh gitu. Sebab saya rasa lo ni ore anggap bendo tu (jangkitan covid) biaso, deme biaso.”  P17 – “Raso situasi lo ni takdok gapo doh sero. Ok dah la. Raso takut dok la, COVID19 takdok dah. Ok lah kito nok gi jalan-jalan, nok gi spita. Nok gi mano-mano tok takut doh lah. Mace takdok COVID19 doh lo ni. Kito cukup doh dengan vaksin tu.”  P17 – “Kalau nok buat tubik (vaksin booster) sekarang pun, takdok ore nok ambik pun sero, mugo COVID19 takdok doh.”  P18 – “Ada lagi ko kume? Rasa OK jah. Maksudnya OK tu, takdok mace dulu lah. Mace (dulu) nak tubik takut kae. Tapi sekarang ni OK lah. Alhamdulillah.  Kalau batuk ko, deme ko, pakai je lah pelitup muko. Sekarang ni COVID19 OK lah daripada dulu, dulu teruk sangat kan. Nok tubik rumoh pun payoh, tapi sekarang ni nak tubik bulih tapi kalau ado batuk ko deme, pakai mask lah.” |
